# The 6 domains of behavior change: the missing health system building block

**DOI:** 10.9745/GHSP-D-13-00083

**Published:** 2013-08-14

**Authors:** James D. Shelton

## Abstract

Behavior is crucial throughout global health interventions. The discipline of behavior change offers distinct expertise needed across 6 different domains of behavior. Such expertise is in short supply, however. We will not have effective and sustainable health systems, nor achieve our ambitious global health goals, without seriously addressing behavior change.

The 2010 Global Burden of Disease (GBD) report makes strikingly clear the absolutely critical role of behavior to health. Despite the limitations of the GBD, it is remarkable that, for sub-Saharan Africa, for example, 15 of the top 20 health risk factors are predominantly behavioral, and the other 5 are highly influenced by behavior (Table).^1^

**TABLE.** Global Burden of Disease 2010: Top 20 Risk Factors, Sub-Saharan Africa

Rank	Risk Factor	Predominantly Behavioral	Strong Behavioral Contribution
1	Alcohol	X	
2	High blood pressure		X
3	Obesity	X	
4	Suboptimal breastfeeding	X	
5	Tobacco	X	
6	High blood glucose		X
7	Household air pollution	X	
8	Diet low in fruits	X	
9	Childhood underweight	X	
10	Iron deficiency	X	
11	Physical inactivity	X	
12	Drug use	X	
13	Diet high in sodium	X	
14	Intimate partner violence	X	
15	Diet low in vegetables	X	
16	Diet low in nuts and seeds	X	
17	Vitamin A deficiency	X	
18	Unimproved sanitation		X
19	High total cholesterol		X
20	Lead exposure		X

Source: Lim et al.^1^

When we think about behavior, we tend to focus on predisposing individual, or “lifestyle,” behaviors with a strong relationship to health and disease. But behavior is crucial throughout health interventions. Indeed, the importance of behavior so permeates all of global health efforts—both preventive and curative—that interventions to influence behavior constitute an overlooked building block in health systems.

Below, I outline 6 domains of behavior and how each is important to our efforts to improve health. Although these domains often overlap, it is useful to consider each domain in turn because they are, to some extent, distinctive. And the principles and skill set of the behavior change discipline can be applied across them.

1. **Freestanding, personal or lifestyle behaviors.** These behaviors, such as those contributing to the risk factors listed in the Table, are behaviors that people can modify on their own, without the involvement of the clinical health system, to improve health or forestall illness. Examples in traditional global health priorities include: exclusive breastfeeding, hand washing, oral rehydration, wearing shoes to prevent helminth infection, and safe sexual practices. But free-standing behavior is also, or, arguably, even more, crucial to prevent noncommunicable disease and injury (NCDI)—behaviors such as tobacco and alcohol avoidance; use of seatbelts, motorcycle helmets, and clean-burning cook stoves; and adequate physical activity. Both over- and undernutrition affect the traditional and NCDI health priorities in numerous profound ways.
2. **Care-seeking behavior or demand.** Clearly, illness often drives people to seek care, but not always. Or, they seek it too late or from the wrong provider. Examples of crucial care-seeking include:
  - Timely access to more skilled delivery services, when complications occur during labor^2^
  - Early treatment of tuberculosis (TB), where it is estimated that the typical person with active TB infects 3 to 6 others^3^
  - Male circumcision for HIV prevention, which has not been readily accepted by older men, who are by far the highest-risk age group and epidemiologically critical^4^
  - HIV counseling and testing, where, unfortunately, many people at risk still forego testing^5^
  - HIV treatment, where many turn to faith healing in search of cure^6^
  - Family planning, where unmet need remains high in many countries^7^
  - Immunization, where “vaccine hesitancy” among parents is a major impediment^8^
  - Treatment of pneumonia and sepsis, especially in the early neonatal period, where recognition of early signs and quick treatment are essential^9^
3. **Client adherence and collaboration.** Once a therapy or other intervention is undertaken, the client's adherence or collaboration can be pivotal. After all, in TB treatment DOTS (Directly Observed Therapy – Short Course) was developed to assure adequate drug adherence. In family planning, discontinuation, especially of oral contraceptives and injectables, is a major reason for unintended pregnancies. In HIV prevention, failure to use condoms correctly and consistently allows infection, while in HIV treatment discontinuation of antiretroviral (ARV) drugs leads to mortality. In malaria prevention, the success of bed nets depends on people, especially the most vulnerable, sleeping under them. Helmets protect only the cyclists who wear them. And clinical approaches to hypertension and diabetes depend greatly on adherence, often to more than one drug. Moreover, poor adherence to antimicrobials leads to drug resistance, which undermines efforts for the entire population.
4. **Provider behavior.** Providers are people, too. We expect them to be competent, caring, and hardworking, but they have values, motivations, misconceptions, capabilities, constraints, social norms, and other priorities like anyone else. What they do and don't do can absolutely make or break many health programs. For example, poor provider performance in IMCI (Integrated Management of Childhood Illness) stems from a variety of behavioral factors including providers' beliefs and misunderstandings, cultural factors, profit motives, negligence, and forgetfulness.^10^ In malaria care, providers' propensity to treat any fever as malaria is dysfunctional.^11^ Many providers continue to treat non-bloody diarrhea with inappropriate antibiotics.^12^ In family planning, many providers avoid IUDs, partly because they lack confidence in their skills and inserting IUDs is time-consuming.^13^ Providers also manage their workloads partly by regulating who and how many clients they see.^13^ And clinicians in general are ill-prepared and ill-disposed to most counseling, despite counseling's often pivotal role.
5. **Pro-social and anti-social behavior.** These are behaviors that influence the health of the community or society at large. They include a wide variety of positive behaviors, such as covering one's mouth when coughing, using latrines, vaccinating one's chickens against avian influenza, and installing traffic-calming devices such as speed bumps, but also negative ones, such as reckless driving, generating air pollution, and even terrorism. These behaviors could be grouped with the first category, the freestanding ones, but typically they require different appeals, transcending appeals to self-interest.
6. **Policy and priority setting.** The behavior of policy makers clearly has a profound influence, notably on funding levels. But programmatic policies are important, too, driving what interventions are prioritized, how they are delivered, the way work is organized, who does what, what gets measured, and what gets rewarded. To some extent, policy is influenced by the medical culture of physicians and other health professionals, who typically manage and influence health programming. A common resulting problem is the preference for curative and clinical services over key prevention interventions. Another is resistance to task shifting, such as provision of simple but vital drugs and contraceptives by community health workers. Similarly, public attitudes are important. Clearly, general concern about HIV has helped galvanize the effective response. In contrast, fatalism and acceptance of maternal mortality as a “normal” occurrence has impeded maternal health initiatives.^14^

**Figure:**
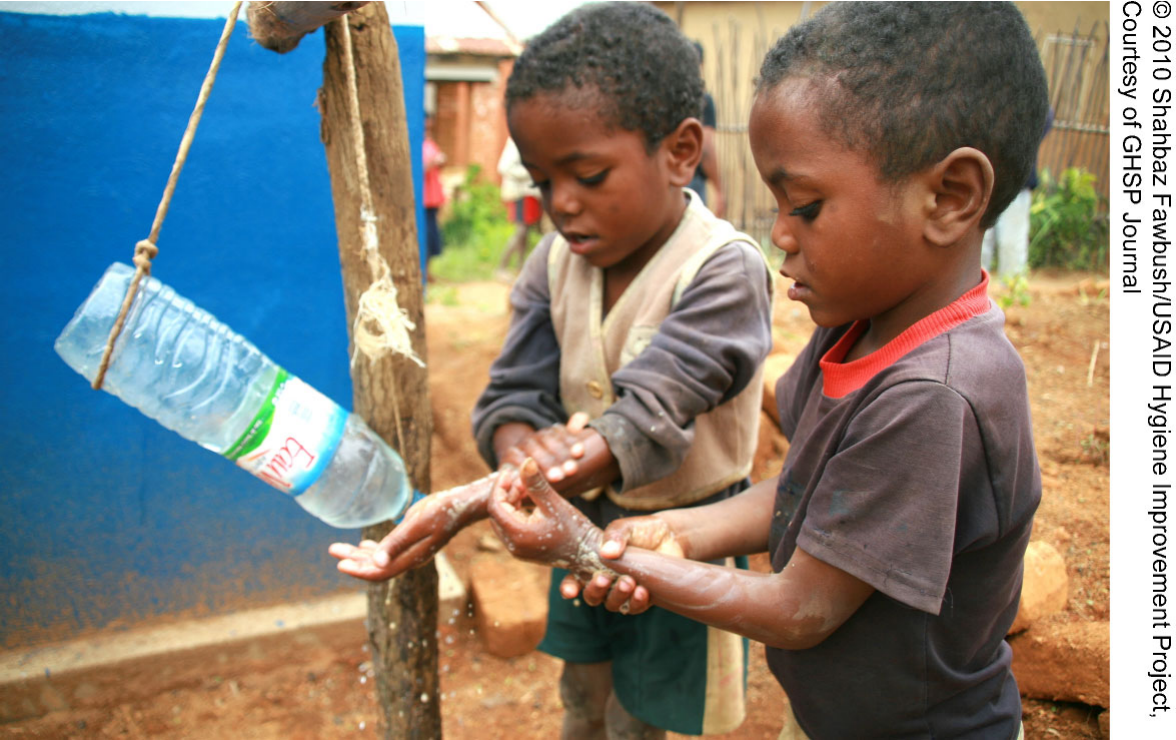
Two boys in Madagascar use soap and a water-saving “tippy tap” to wash their hands.

## APPLYING COMMON PRINCIPLES OF BEHAVIOR CHANGE ACROSS THE DOMAINS

Why is appreciating these domains important? First, success in many global health priorities requires addressing multiple domains collectively. For example, good “combination” prevention in HIV calls for reducing sexual risk-taking and correct use of condoms (domains 1, 3, and 5), initiation and adherence to ARV drugs (since ARVs inhibit transmission) (domains 2 and 3), good counseling to support these behaviors among clients (domain 4), building demand for male circumcision and HIV testing (domain 2), and adequate funding and enlightened policies such as task shifting to support all of it (domain 6). In this issue of GHSP, Kamhawi et al. address 3 domains—promoting client demand (2), improving provider counseling (4), and supporting continued successful use (3)—in their intervention to improve use of family planning.^15^

At the same time, although each domain has its own characteristics, behavior change is a distinct discipline with its own approaches and skill sets that apply across the domains. Briefly, its components include:

- Understanding thoroughly the intended audiences, often through formative research
- Tailoring approaches to those audiences
- Using multiple vehicles of messaging and learning
- Appealing effectively to both heart and mind
- Promoting sustained behavior change, including building health literacy and positive habits
- Addressing key family members and communities and the social norms that they help to establish
- Including structural approaches that support positive behavior (for example, tobacco tax, phone text medication reminders, and performance checklists for providers)
- Addressing incentives
- Measuring impact and making adjustments on a continuing basis

We can apply this behavioral expertise across all 6 domains and promote synergy. But behavioral expertise is in precious short supply, especially in developing countries. We need to expand and strengthen that expertise in institutions and to foster competent behavior specialists, but also to increase the behavioral expertise of all health cadres.

Any health system worth its salt must address the missing building block of behavior change in a vigorous way. We cannot achieve our ambitious global health goals without it.
